# Suspended Solid-state Membranes on Glass Chips with Sub 1-pF Capacitance for Biomolecule Sensing Applications

**DOI:** 10.1038/srep17775

**Published:** 2015-12-08

**Authors:** Adrian Balan, Chen-Chi Chien, Rebecca Engelke, Marija Drndić

**Affiliations:** 1 Department of Physics and Astronomy, University of Pennsylvania, Philadelphia, Pennsylvania, 19104, USA

## Abstract

Solid-state membranes are finding use in many applications in nanoelectronics and nanomedicine, from single molecule sensors to water filtration, and yet many of their electronics applications are limited by the relatively high current noise and low bandwidth stemming from the relatively high capacitance (>10 pF) of the membrane chips. To address this problem, we devised an integrated fabrication process to grow and define circular silicon nitride membranes on glass chips that successfully lower the chip capacitance to below 1 pF. We use these devices to demonstrate low-noise, high-bandwidth DNA translocation measurements. We also make use of this versatile, low-capacitance platform to suspend other thin, two-dimensional membrane such as graphene.

Many personalized medicine, environmental, and mechanical applications require the use of membranes that could effectively and reliably separate two regions of empty space or fluids. Several thin membranes, sometimes as thin as one atom in the case of graphene, have proven impermeable to gas or fluid flow unless nanoholes are introduced into them^1^. In the presence of nanoholes, molecular flow can be controllably driven through them and this flow is maximized as the membrane thickness is reduced, creating new opportunities for optimizing either the ionic current signals for genetic sequencing^2^,^3^,^4^, the water flux for filtration and desalination^5^, or the sensitivity to molecular structure in nanoscale devices exploiting freestanding thin membranes^6^ . Progress in the field has been fast, in large part thanks to the development of new experimental techniques that enable unprecedented control at the nanoscale to grow and place membranes at desired positions relative to a substrate^2^. The past few years have witnessed significant results in developing such membranes and using them in applications ranging from mechanics^1^ to nanoelectronics^7^ and biomedicine^8^.

In many biomedically-relevant nanoelectronics applications, it is advantageous that the overall capacitance of the membrane chip is small in order to minimize the electrical noise produced when the voltage noise from the power source couples to the total capacitance in the system. Frequently, the chip capacitance is the dominant capacitance and it governs the lowest current noise that can be achieved at a particular bandwidth^9^. Specifically, this is important for the use of membranes for biomolecule detection^8^, a class of applications attracting increasing interest and witnessing significant progress. Solid-state membrane devices with nanopores have been used for example to differentiate biomolecules such as proteins^10^,^11^,^12^, DNA homopolymers^13^ and miRNA^14^, to determine nanoparticle surface charges^15^, or as nanoscale reactors for nanoparticle synthesis^16^. However, the wide applicability of these results is limited by the noise in the ionic current signal. Several approaches have been explored to improve the signal-to-noise ratio, either by reducing the nanopore size^13^ and thickness^17^, or by placing a nanoribbon next to the nanopore as a sensor^6^.

Above 10 kHz frequency, the noise largely results from the interaction between the amplifier’s voltage noise and the chip capacitance. I_rms_, the root-mean-square input referred current noise, is given by the equation 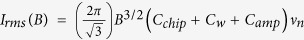
9. Here, B is the bandwidth, *v*_*n*_ is the input-referred voltage noise of the amplifier, C_chip_ is the chip capacitance, C_w_ is the capacitance of the wiring from the amplifier to the chip and C_amp_ is the capacitance of the amplifier. Therefore, one major approach to improve the noise, and consequently the signal-to-noise ratio, is to reduce the chip capacitance. There have been several efforts to reduce the chip capacitance by bonding glass slides onto the silicon chip^9^, or transferring silicon nitride membranes onto glass substrates^18^,^19^. The thin silicon nitride membranes on glass chips reported here further reduce the chip capacitance to below what has been reported previously^9^,^13^,^18^,^19^. The reported fabrication process is also transfer-free and therefore, chips can be made quickly and in large quantities. Besides, these chips are suitable to the harsh acid or plasma treatments often used to make chips hydrophilic or to clean the chips for reuse between experiments.

In this paper, we report the design and fabrication of chips consisting of suspended silicon nitride membranes spanning over apertures on glass substrates. We introduce two new chip designs: a simple chip design that routinely results in sub-2 pF capacitances, and also an improved two-step fabrication design resulting in sub-1 pF capacitance chips. The main innovation is to replace the conventional design silicon substrate which contributes to most of the device capacitance with an insulating glass substrate. The resulting total chip capacitance below 1pF leads to an improved noise performance in ionic current signals^9^ that we illustrate by low noise DNA translocation experiments, paving the way to more sensitive measurements for biomolecules detection and differentiation. The fabrication process for silicon nitride membranes on glass reported here is transfer-free and suitable for large-scale production. We also demonstrate the use of this platform to suspend other 2D materials (graphene) on top of holes in silicon nitride membrane that are in turn suspended across the aperature.

## Results

**Figure 1:**
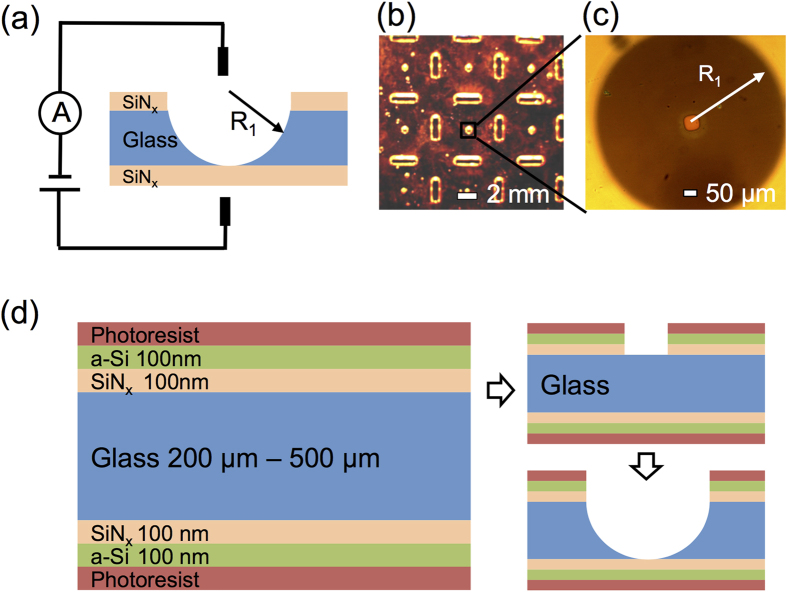
(**a**) Device schematic of a glass chip with a suspended silicon nitride membrane positioned between two chambers of electrolyte solution with bias voltage applied across the membrane. (**b**) Optical image of the glass wafer containing several 5 × 5 mm^2^ glass chips. (**c**) An optical micrograph of the center area of the glass chip. The SiN_x_ membrane is freestanding at the center of glass isotropically etched away forming a hollow sphere (grey circle shadow). (**d**) Schematic of the glass and the membrane manufacturing process. On both sides of the 200–500 μm thick glass we deposited 100 nm of SiN_x_ and 100 nm a-Si. We spin-coat 10 μm of SPR-200 photoresist on both sides. Squares of 25 μm in size are patterned using photolithography at the center, and then the a-Si and SiN_x_ layers are removed with reactive ion etching. The glass is then etched in 49% HF solution until the sphere created by isotropic etching reaches the bottom of the SiN_x_ layer. The remaining photoresist and a-Si are stripped away, forming the aperture in the glass chip covered by the silicon nitride membrane.

Figure 1a shows the measurement setup for the low-capacitance devices in electrolyte solution. The silicon nitride membrane is supported by a glass chip, 5 mm × 5 mm wide and 200–500 μm thick. Typically a voltage up to 1 V is applied across the membrane with an amplifier (Chimera Instruments, New York, NY) to drive the analytes in solution to interact with the membrane or pass through the nanopore. By measuring the resulting current variations, nanopores have been shown to differentiate biomolecules with various sizes, structures, and charges^8^,^10^,^11^,^12^,^13^,^14^,^20^,^21^. Two images of glass chips at different magnifications are shown in Fig. 1b,c. Figure 1c shows the silicon nitride (SiN_x_) membrane suspended across a central aperture, several micrometers in size, where glass is etched away. Figure 1d demonstrates the fabrication process of the SiN_x_ membranes on glass chips. On both sides of the glass wafer, we employ an LPCVD technique to deposit a layer of 100-nm-thick SiN_x_ as the membrane material and 100-nm-thick amorphous Si (a-Si) layer on top of SiN_x_ as a protection against the hydrogen fluoride (HF) etch later in the process. The glass used in this study is fused silica, which has a high softening point allowing the LPCVD process for high quality SiN_x_ membranes. We spin-coat SPR-220 photoresist on both sides, and we pattern by photolithography squares of 10 μm in size at the center and etch away the a-Si and SiN_x_ layers by CF_4_ reactive ion etching. The glass substrate is etched in a 49% HF solution until the sphere created by isotropic etching reaches the bottom SiN_x_ layer. The remaining photoresist and a-Si are stripped away by acetone and KOH respectively, resulting in the final glass chip with a silicon nitride membrane suspended at its center.

**Figure 2:**
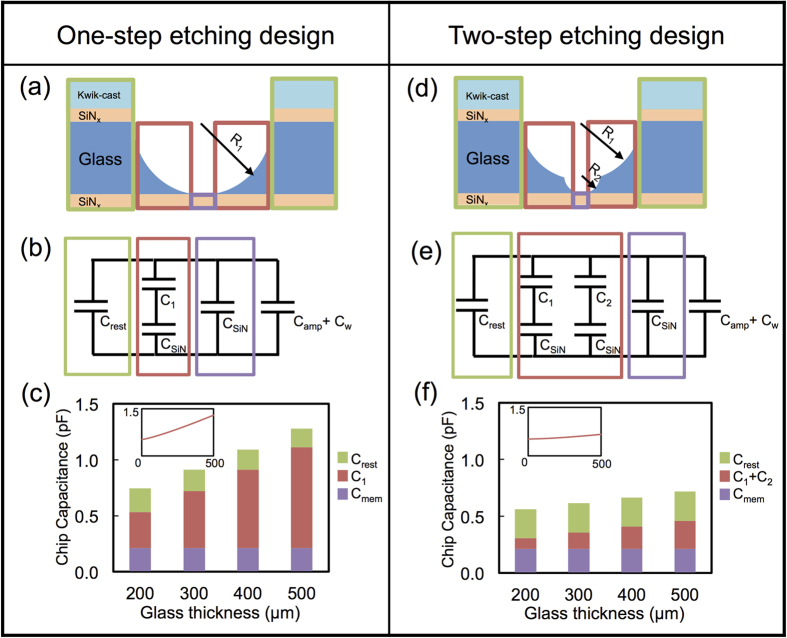
(**a**) Schematic of the glass chip with silicon nitride membrane in the center, with silicone elastomer (Kiwk-cast) applied on top as insulation. The different areas contributing to the chip capacitance are color-coded. C_mem_ is the capacitance of the membrane, C_1_ is the capacitance of the spherical area, and C_rest_ is the capacitance of the rest of the chip. (**b**) The corresponding circuit diagram, where C_mem_, C_1_, C_rest_ are capacitances of the purple, red, and green area respectively. (**c**) Bar graph of chip capacitance for glass thickness of 200, 300, 400, and 500 μm, showing relative contributions from regions C_rest_, C_1_ and C_mem_. The inset is the minimum capacitance as a function of glass thickness. The units are the same as the main figure. (**d**) Schematic of the glass chip device produced by a two-step etching; the first etched sphere has a radius R_1_, and the second etched sphere has a radius R_2_. The different areas contributing to the chip capacitance are color-coded. C_mem_ is the capacitance of the membrane, C_1_ is the capacitance of the spherical area with radius R_1_, C_2_ is the capacitance of the spherical area with radius R_2_, and C_rest_ is the capacitance of the rest of the chip. (**e**) The corresponding capacitor circuit diagram, where C_mem_, C1 + C_2_, C_rest_ are capacitances of the purple, red, and green areas, respectively. (**f**) Bar graph of the chip capacitance for glass thickness of 200, 300, 400, and 500 μm, respectively, showing relative contributions from regions C_rest_, C1 + C_2_ and C_mem_. The inset is the minimum capacitance as a function of glass thickness. The units are the same as the main figure.

In order to estimate the total capacitance of the resulting glass chip, we represent the glass chip as a set of individual capacitive elements and calculate each capacitance contribution to the total chip capacitance as shown in the color-coded regions in Fig. 2a. Each color-coded area represents parallel capacitors and could be summarized by a circuit diagram as shown in Fig. 2b. The purple area is the freestanding SiN_x_ membrane with capacitance C_mem_. The red area is the area where the glass substrate was etched away isotropically, creating a hemispherical structure. The capacitance in this area is estimated by integrating infinitesimal parallel plates over the whole sphere area and is given by
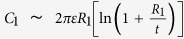
, where R_1_ is the thickness of the glass chip and also the radius of the sphere, t is the thickness of the SiN_x_ layer, and 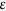 is the permittivity of glass. The green area is the remaining area of the chip covered by a layer of silicone elastomer (Kwik-cast, World Precision Instruments) as a seal to separate the two chambers of electrolyte solutions, and its capacitance is noted as C_rest_. The estimated total chip capacitance is summed up to be C_mem_ + C_1_ + C_rest_.

Figure 2c summarizes the results of the estimated total chip capacitance with glass thickness of 200 μm to 500 μm. Here we assume a membrane radius of 10 μm, with 0.5 mm Kwik-cast applied around the etched sphere, and an electrolyte solution droplet covering an area ~7 mm^2^ (1.5 mm radius of circular area coverage) serving as electrolyte chamber. Glass chips designed by this method can reach capacitances below 1 pF for glass thickness below 300 μm. We note that the relatively large contribution to the overall capacitance comes from the red area in Fig. 2d where the dielectric layer is the thinnest.

In order to further lower the total chip capacitance, we refine this fabrication process to incorporate a two-step etching method, obtaining the chip structure shown in Fig. 2d. In this two-step process we stop the HF etching when the etched sphere (Fig. 2d) approaches the SiN_x_ layer at a distance of several micrometers. The main idea is to leave as much of the insulating material close to the center, while still being able to define a suspended membrane of desired size. We spin on the electron beam resist (ZEP520) and perform electron beam lithography to create a patterned aperture smaller than 5 μm in radius. The whole chip is then etched again in the HF bath until the second etched sphere reaches the SiN_x_ layer and forms a suspended membrane. Similar to the previous circuit diagram (Fig. 2b), we calculate the total chip capacitance of this chip structure by dividing it into capacitors represented by different color-coded regions. The main difference between the two designs is that in this two-step etching process, there are now two etched spheres stacked on top of each other. The capacitance contribution of the red area is now given by 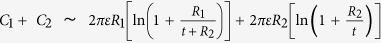, where R_1_ is the radius of the upper sphere, and R_2_ is the radius of the lower sphere. The sum of the radii of the two spheres is equal to the total thickness of the chip. The capacitor circuit diagram of this chip design is shown in Fig. 2e. With this chip design, the theoretical minimum of the total chip capacitance for glass in the 200–500 μm thickness range could be reduced by approximately 0.5 pF compared to that of chips produced by one-step etching. In this case, the contribution from the red area where the glass is etched away in a spherical shape is greatly reduced (Fig. 2f, and Fig. S1). This reduction in capacitance is due to a thicker insulating glass layer remaining in the chip. It is useful to estimate the capacitance minimum as a function of the device thickness as shown in the inset of Fig. 2c,f. In both designs, the capacitance asymptotically approaches a minimum value of 0.5 pF given by the large Kwik-cast covered area when the glass thickness and sphere radius approaches zero. However, the capacitance of the two-step etching design is much less sensitive to the membrane thickness, allowing us to use more robust 300 μm glass chips in our experiments.

**Figure 3:**
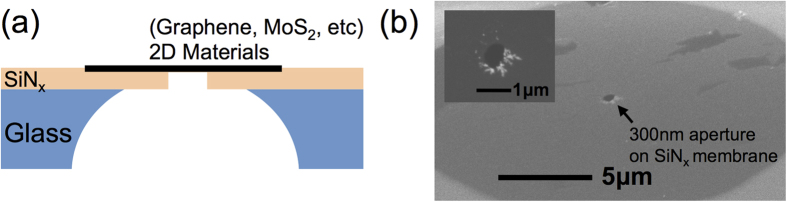
(**a**) 2D materials, such as graphene or MoS_2_, on the SiN_x_-on-glass chip serving as a sub- 1 pF capacitance platform. (**b**) SEM image of a graphene sheet covering the 300-nm-large aperture in the suspended SiN_x_ membrane. The darker grey area is the circular SiN_x_ membrane, and graphene is covering most of the area of the SiN_x_ membrane, including the aperture at the center. The inset is the enlarged image of the aperture with graphene suspended on top. The white flakes around the suspended graphene area(circular dark area in the center) are residual PMMA after transfer. A large-view of the whole SiN_x_ membrane with the graphene suspended at the center is included in Fig. S3.

Realizing nanopores in ultrathin membranes is particularly important for two reasons: to maximize the ionic current signal level (by minimizing the nanopore thickness and therefore its resistance), and to maximize the spatial sensitivity (in order to sample a small part of the molecule, such as a single DNA base). One straightforward approach to realize ultrathin membranes would be to thin the SiN_x_ membrane by reactive-ion etching^13^ or STEM thinning^17^ until we reach the stability limit of a-Si around 1 nm^16^. Alternatively, we can use these glass chips as a more general platform to suspend 2D materials such as graphene (3.4 Å thick) and metal dichalcogenides (MoS_2_, WS_2_ ~ 6 Å thick) monolayer membranes. 2D materials due to its atomic-level thickness have been shown to be promising biomolecule sensors^2^,^3^,^4^,^22^,^23^. The low capacitance SiN_x_-on-glass platform shown in Fig. 1a will concur to enhance the sensing capabilities of such 2D materials. Figure 3a is a schematic illustrating a 2D membrane suspended over a ~300-nm-size aperture formed in the SiN_x_ membrane over the glass chip. One example is the scanning electron microscope (SEM) image of a graphene sheet suspended in this fashion (Fig. 3b). The 300-nm-size aperture in the SiN_x_ membrane was first fabricated by electron beam lithography and plasma etching. CVD grown graphene characterized by Raman spectroscopy (Fig. S4) was then transferred^24^ onto the membrane over the aperture. The device is placed into electrolyte solution as shown in Fig. 1a, and when bias voltage is applied the current flowing through is nearly zero suggesting complete graphene coverage over the aperture. In principle, other 2D materials, such as metal dichalcogenides monolayer membranes could be transferred onto this SiN_x_-on-glass platform as typically transferred onto Si substrate^25^. Gurarslan *et al.*^25^ have demonstrated the transfer of MoS_2_ layer onto arbitrary substrate, creating the possibility to extend the usefulness of this SiN_x_-on-glass chip design to MoS2 and likely other 2D materials.

**Figure 4:**
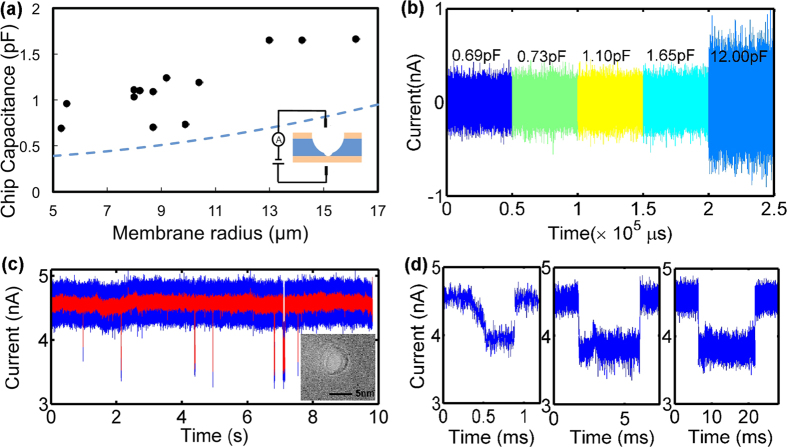
(**a**) Measured capacitance, C_chip_, of the glass chips produced by two-step etching (Fig. 2d) as a function of SiN_x_ membrane radius (μm). The blue dashed line is C_chip_ estimated from the model in Fig. 2f. The glass chip thickness is 300 μm and the SiN_x_ membrane thickness is 100 nm. The experimental errors are within the area of the solid circles. (**b**) Measured ion current temporal traces for several glass chips with capacitances C_chip_ = 0.69, 0.73, 1.1 pF, and 1.65 pF showing an amplifier-limited noise. A current trace from a 12 pF is shown for comparison (**c**) Current vs. time trace of 3 kbp (kilo base pairs) dsDNA segments translocating through one of the devices. The red trace is filtered at 100 kHz, and the blue trace is filtered at 1 MHz. The inset is a TEM image of a nanopore drilled with focused electron beam in the TEM. (**d**) Details of events with lengths from 0.5 ms to 10 ms from Fig. 3c.

Measured chip capacitance, C_chip_, for 13 devices are summarized in Fig. 4a as solid circles, and the dashed blue line is the estimated minimum attainable capacitance. The chips are made with fused silica of thickness 300 μm and SiN_x_ membrane thickness 100 nm, fabricated by the two-step etching method described previously. Chip capacitance was measured by applying triangular-wave voltage pulses with the Chimera amplifier and measuring current *vs.* time in a fluidic cell as shown in Fig. 1a. For membranes with radii smaller than 10 μm, we experimentally attain C_chip_ in the sub-1 pF regime, and we observe that C_chip_ scales with the membrane radius in accordance with our estimation. The experimental values are slightly larger than the theoretical estimate because of the actual radius of the second sphere is smaller than the radius R_2_ (Fig. 2d), which was used to estimate the minimum capacitance based on a two-sphere representation in Fig. 2f. This is largely caused by ZEP520 electron beam resist not being able to withstand long HF etch and flaking off before the desired etch time. Other possible factors include errors in estimates of the thickness of the silicon elastomer Kwik-cast and its distance to the membrane, and the size of the liquid droplet.

When the capacitance of chips is on the same order of the internal amplifier capacitance (~20 pF), we observe the contribution of the chip capacitance to the current noise (here shown for a 12 pF device in Fig. 4b). As the capacitances of these glass chips are much lower than the amplifier internal capacitance, the resulting ionic current noise we observed for these chips shown in Fig. 3b is undistinguishable from the open-headstage noise (~110 pA_rms_ ). In other words, in this case the noise is dominated by the amplifier^9^.

The silicon nitride membrane chips demonstrated here are interesting for applications requiring low capacitances, including the realization of nanopores for biomolecule analysis and DNA sequencing. We use these devices to demonstrate very low noise translocations of 3 kbp double-stranded (ds) DNA. Nanopores can be drilled, for example, with a focused electron beam in transmission electron microscope (TEM)^9^,^12^,^13^,^14^,^15^,^16^,^17^or by controlled dielectric breakdown^26^. A TEM image of a nanopore drilled by focused electron beam on 100 nm thick SiN_x_ membrane is shown in the inset of Fig. 4c. Figure 4c (and Fig. S2a) shows an ionic current *vs.* time trace of translocation events measured at 1 MHz (blue trace) and filtered at 100 kHz (red trace) for 1 V bias voltage, and Fig. 4d shows details of events with lengths from 0.5 ms to 10 ms. The total noise compares favorably to previous measurements at the same frequency^9^,^13^,^14^, despite the higher applied voltage. Also, using these low capacitance chips and a high frequency amplifier we are able to detect small (180 bases), single stranded DNA segments with better noise characteristics than in previous result^12^ (Fig. S2b).

## Discussion

In summary, we designed an integrated process of producing membranes on glass chips with sub-1 pF capacitance and we demonstrated their use in DNA translocation experiments. To achieve DNA sequencing using solid-state nanopores without slowing down the molecules, we have to be able to measure the ionic current at frequencies close to 20 MHz^9^. Other biomolecules sensing applications utilizing nanopores also require to be performed at high bandwidth with enhanced signal to noise ratio to differentiate finer features of the biomolecules^12^. To achieve this goal we need to ameliorate the signal level by decreasing the membrane thickness and pore size^12^, design new low noise high bandwidth amplifiers^9^,^12^ and develop devices with capacitances smaller than 1 pF. This work has shown significant improvement on reducing the chip capacitance by substituting glass for silicon as a substrate. One-step fabrication process could produce sub-2 pF capacitance and further sub-1 pF capacitance was realized by an improved two-step design, resulting in a low-noise device for biomolecules detection demonstrated by DNA translocation experiments. The lowered noise level and the ease of production and cleaning make these glass chips superior substitutes for conventional silicon chips. Furthermore, this versatile platform could, in principle, be used to suspend 2D materials to achieve higher signal to noise levels in various nanoelectronics and biomolecule detection applications^2^,^3^,^4^,^22^,^23^.

## Methods

Device fabrication: The 4 inch glass wafer consists of fused silica thickness of 200–500 um, and SiN_x_ thickness of 100 nm was deposited on both sides with LPCVD. A layer of 100 nm of a-Si is also deposited by the same technique. We spin-coat SPR-220 photoresist on both sides, and a chromium mask with 10 um window and dividing lines was used to pattern squares of 10 μm in size at the center and divide the wafer into 5 × 5 mm chips by photolithography. The a-Si and SiN_x_ layers are etched away by reactive-ion etching using CF_4_ gas. The glass substrate is etched in a 49% HF solution with an etch rate ~1 μm/min until the sphere created by isotropic etching reaches the bottom SiN_x_ layer. The remaining photoresist and a-Si are stripped away by acetone and KOH respectively.

For the two-step etching design, we spin-coat on the electron beam resist (ZEP520) covering the entire chip and perform electron beam lithography to create a patterned aperture smaller than 5 μm in radius at the center of the etched sphere. The chip is then etched again in the HF bath until the second etched sphere reaches the SiN_x_ layer and forms a suspended membrane.

Single-layer CVD graphene was grown on copper foil, and could be cut into squares to fit the size of the chip. After spinning on a PMMA support layer we use the bubbling transfer techniques to isolate the graphene and PMMA from copper^27^. The graphene is then transferred onto the glass chip with a 300 nm aperture into the suspended SiN membrane. The quality of CVD-grown graphene is discussed in supplemental information and characterized by Raman spectroscopy (Fig. S4)^28^,^29^ and its defect concentration is estimated as in Gogneau *et al.*^30^.

Nanopores are drilled using a focused electron beam on a JEOL 2010F transmission electron microscope operating at 200 kV.

Measurements: The measurement cell has two chambers of 1 M KCl, 1 mM EDTA solution buffered using 10 mM TrisHCl. Experiments were conducted using a VC100 voltage-lamp amplifier (Chimera Instruments, New York, NY), to apply the bias voltage using Ag/AgCl electrodes and measure the ion current through the nanopore. The amplifier applies a fourth order Bessel low-pass filter at 1 MHz. For translocation experiments bias voltages of 200 mV−1 V are applied across the nanopore. For capacitance measurements a triangle wave is applied and the capacitance is estimated from the current variation when the sign of the voltage slope changes, similar to measurements done by Balan *et al.*^9^

## Additional Information

**How to cite this article**: Balan, A. *et al.* Suspended Solid-state Membranes on Glass Chips with Sub 1-pF Capacitance for Biomolecule Sensing Applications. *Sci. Rep.* **5**, 17775; doi: 10.1038/srep17775 (2015).

## Supplementary Material

Supplementary Information
