# Supplementary material for: Suspended Solid-state Membranes on Glass Chips with Sub 1-pF Capacitance for Biomolecule Sensing Applications
Source: Sci Rep. 2015 Dec 8;5:17775. doi: 10.1038/srep17775 (PMC4672352; doi:10.1038/srep17775)
Supplement: Supplementary Information [file srep17775-s1.pdf]

Supplementary information

Suspended Solid-state Membranes on Glass Chips with Sub 1-pF Capacitance for Biomolecule Sensing Applications

Adrian Balan[1,+], Chen-Chi Chien[1,+], Rebecca Engelke[1], and Marija Drndic [1,*]

Department of Physics and Astronomy, University of Pennsylvania, Philadelphia, Pennsylvania,

19104, USA

*drndic@physics.upenn.edu

[+]these authors contributed equally to this work

S1. Minimum capacitance for the two designs compared

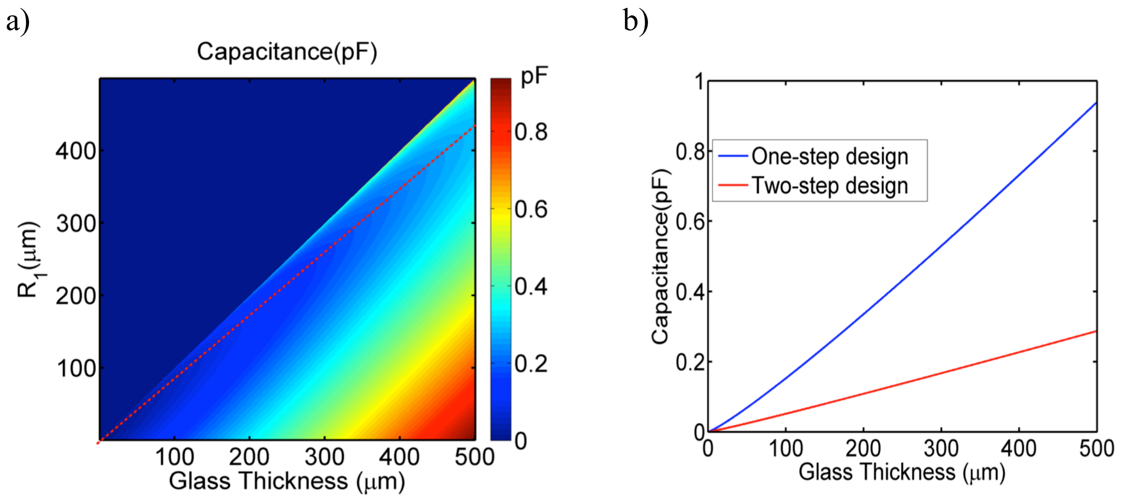

Figure S1. a) Capacitance of the spherical part of the two-step design as a function of the glass slide thickness and radius of the bigger sphere (R_1). The red dotted line indicates the R_1 at which the minimum of the capacitance is found. b) Comparison of the minimal capacitance as a function of the device thickness for the spherical part (red area in Fig. 2a and 2d) of the two designs.

S2. Improved noise for DNA translation measurement

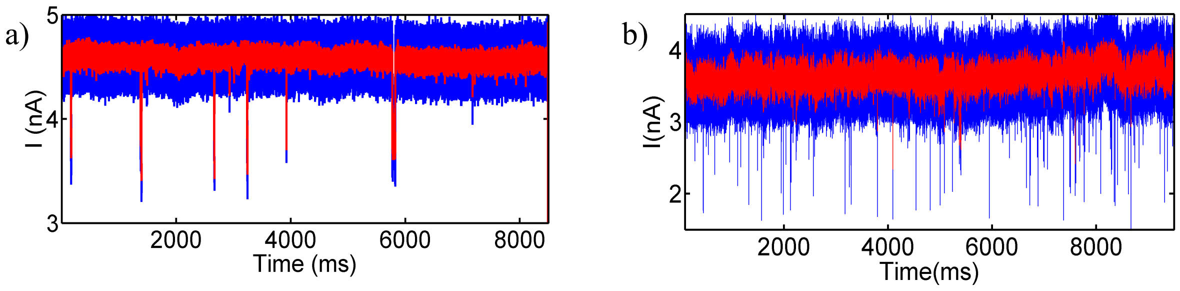

Figure S2. a) 3kbps dsDNA segments translocating through the same 1pF device as in figure 4c. b) Detection of short single stranded DNA segments (180bp) through a device with a nanopore of approximately 3nm in diameter. The red trace is filtered at 100kHz, and the blue trace is filtered at 1MHz.

S3. SEM image of graphene suspending over aperature on SiN_x-on-glass device

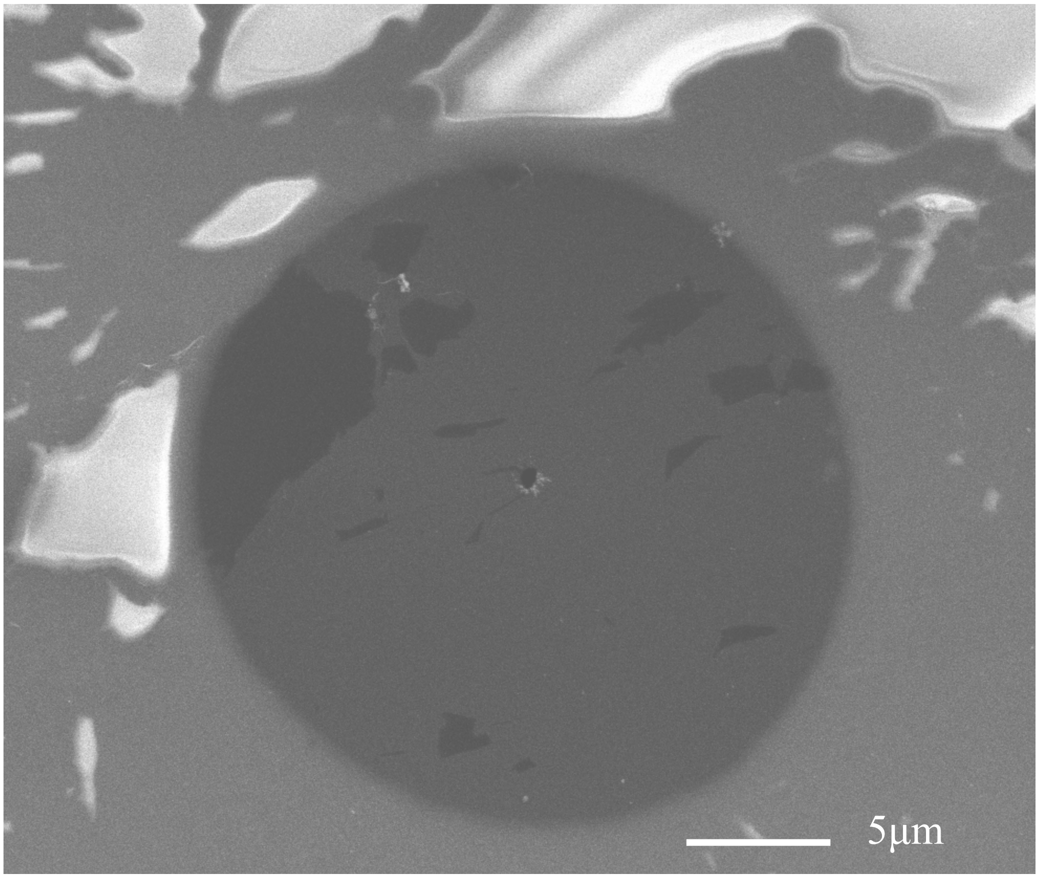

Figure S3. An SEM image of the large-scale view of the membrane, the 300nm aperture, and graphene coverage. We could identify that graphene covers over the entire aperature and most of the area shown. The part that is not covered by graphene is showing significant charging due to the insulating glass substrate.

S4. Graphene characterization

The defect spacing can be estimated from the Raman spectra. In Figure S4 a) we present one of the characteristic Raman spectra from the cartography we did on one generic Graphene sample after transfer to the SiN suspended membrane. The background coming from the SiN membrane is already subtracted. We identify the Raman signature of graphene (the peak G at 1580cm^{-1} at 2D at 2680cm^{-1}). A faint D peak, defect activated, can be seen around 1340cm^{-1}. Its integrated amplitude represents only 5% to 10% of the amplitude of the amplitude of the G peak. Figure S4 b) present the ratio I(D)/I(G) in all the sample. Similar to [Gogneau *et al.*][1] , we estimate the defect spacing the empirical formula L = [2.4x10^{-10} nm-3]$\lambda^4(I_G/I_D)$, where λ = 532 nm is the excitation wavelength (green laser) , and I_D and I_G are the integrated intensities of the G and 2D peaks. We obtain defect spacing between 200 and 300nm, comparable to the diameter of suspended graphene area in this paper. This low concentration of defects explains why we obtain no ionic conductivity in this intact membrane, similar to results obtained previously on graphene membranes that should be not permeable to water or gases (except H_2).

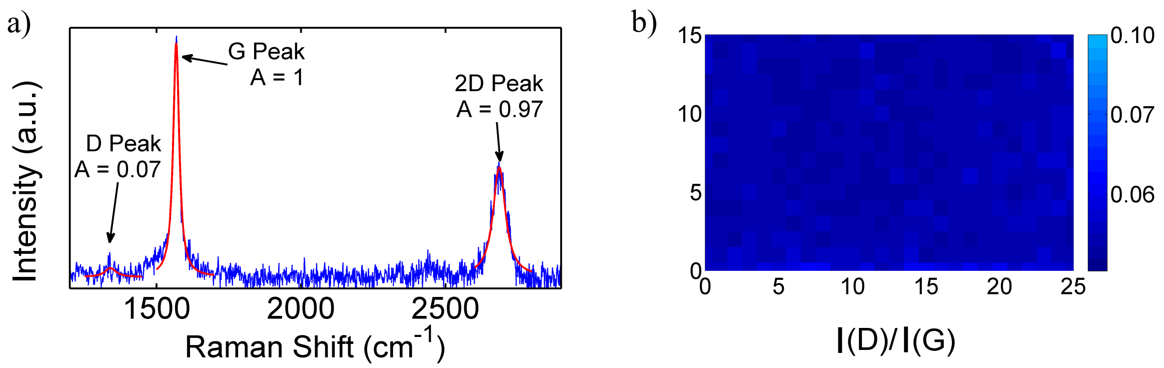

Figure S4. a) Representative Raman spectra acquired on a graphene sample transferred to a SIN membrane. The integrated intensities of the peaks D, G, and 2D, normalized to the intensity of the G peak is also presented (A= 0.07 , 1, 0.97 respectively for the D,G and 2D peaks) b) Map of the ratio of the integrated intensities of the D and G peaks. Each point in the Raman map is 500nm.

1. Gogneau, N., Balan, A., Ridene, M., Shukla, A. & Ouerghi, A. Control of the degree of surface graphitization on 3C-SiC(100)/Si(100). *Surf. Sci.* **606,** 217–220 (2011).
